# Synthesis of Thermoplastic Polyurethanes Containing Bio-Based Polyester Polyol and Their Fiber Property

**DOI:** 10.3390/polym14102033

**Published:** 2022-05-16

**Authors:** Jiyeon Oh, Young Kwang Kim, Sung-Ho Hwang, Hyun-Chul Kim, Jae-Hun Jung, Cho-Hyun Jeon, Jongwon Kim, Sang Kyoo Lim

**Affiliations:** 1 Division of Energy Technology, Daegu Gyeongbuk Institute of Science and Technology (DGIST), Daegu 42988, Korea; ojy0923@dgist.ac.kr (J.O.); kimyk1211@dgist.ac.kr (Y.K.K.); hsungho@dgist.ac.kr (S.-H.H.); 2 Division of Biotechnology, Daegu Gyeongbuk Institute of Science and Technology (DGIST), Daegu 42988, Korea; kimhc@dgist.ac.kr; 3 Living Materials Research Lab, Korea Textile Development Institute (KTDI), Daegu 41842, Korea; jhjung@textile.or.kr; 4 Eco-Friendly Materials Team, Korea Textile Development Institute (KTDI), Daegu 41842, Korea; adjch@textile.or.kr; 5 Department of Fiber System Engineering, Yeungnam University, Gyeongsan 38541, Korea; kjwfiber@ynu.ac.kr; 6 Department of Interdisciplinary Engineering, Daegu Gyeongbuk Institute of Science and Technology (DGIST), Daegu 42988, Korea

**Keywords:** biobased material, one-shot polymerization, thermoplastic polyurethane fiber, physicochemical properties

## Abstract

Among the starting materials of thermoplastic polyurethanes (TPUs), it was confirmed that succinic acid-based polyester biopolyols having different molecular weights (M_n_ = 1000, 2000, and 4000) affect the physicochemical properties of the final polymer significantly. Bio-TPUs synthesized through a solvent-free one-shot polymerization process were synthesized with a polyester polyol, 1,4 butanediol (BDO), and 4,4′-methylene diphenyl diisocyanate (MDI) in a molar ratio of 1:1:2. As a control group, one typical petroleum-based TPU was synthesized and characterized along with other bio-based TPUs. Representative petroleum-based and bio-based TPUs synthesized were manufactured as monofilaments with a diameter of about 0.2 mm through an extrusion process with different draw ratios (4, 5, and 6 times). The molecular weight and structural properties of the TPUs were characterized by GPC and FT-IR analysis and thermal characterization by DSC and TGA analysis. Petroleum-based TPU and bio-based TPU having the same molecular weight soft segment (SS) tended to have similar molecular weight and hard segment (HS) content. TPUs with high HS content had excellent thermal stability, enabling stable extrusion of TPUs. In addition, it was confirmed that the bio-based TPU fibers produced in this way had a tensile strength corresponding to the physical properties of petroleum-based TPU fibers and an excellent elastic recovery rate of almost 100 %. These results indicate the application potential of bio-TPU.

## 1. Introduction

Thermoplastic polyurethanes (TPUs) are one of the most consumed families of polymers in the world. They are used as components in fibers, paints, coatings, foams, adhesives, and packaging in numerous fields such as the automotive industry, consumer or domestic equipment, construction engineering, and biomedical application [1,2,3,4]. 

TPUs are liner segmented block copolymers composed of soft segments (SS) and hard segments (HS), which undergo phase separation [5,6]. The factors which influence the phase separation include segmental polarity difference, segmental length of either segment, intra- and intersegment interactions such as hydrogen bonding, overall composition, and molecular weight [7]. The HS is typically made of a rigid diisocyanate and a chain extender (e.g., a diol), whereas the SS mainly consists of a long polyol. Such systems usually segregate into microphases or domains [8]. Different parameters, e.g., the molar mass of the long polyol and the concentration of HS in the polymeric matrix, permit finely tailoring and controlling the physicochemical and mechanical properties of the final TPUs [9,10]. The physical properties of TPUs are affected by the degree and shape of phase separation, which is strongly related to HS content [11]. As the molecular weight of polyols used for TPUs production increases, the fine phase separation of the soft and hard parts tends to occur. In general, the higher the molecular weight of polyols, the lower the HS content, so the polyol has a morphology with an isolated association region. On the other hand, when TPUs are prepared with polyols having a low molecular weight, HS has a characteristic morphology, in which HS has an association region connected [12,13,14]. These structural changes have a profound effect on the resulting physical properties.

Furthermore, the polymerization methods, i.e., one-shot and prepolymer methods, can affect the length and distribution of HS at a fixed monomer feed ratio [15]. In the one-shot method, all the monomers are fed into the reactor simultaneously, and oligomeric diol and short-chain diol compete to react with diisocyanate. Thus, when the short-chain diol has higher reactivity than the oligomeric diol, some longer HS and an accompanying wider distribution of HS lengths are obtained [16]. Prominently, one-shot polymerization is commonly used in industrial applications. Solvent-free and one-shot polymerization synthesis of TPUs have substantial theoretical and practical value [17]. In particular, there are more environmental advantages than the conventional solvent-based synthesis methods. However, the solvent-free synthesis of TPUs has been less studied than the synthesis of solvent-based and waterborne TPUs [18]. According to Wilkes et al. [15] and Cho et al. [16], the broader distribution of HS lengths in TPUs prepared by the one-shot method compared with that prepared by the prepolymer method was observed [19]. 

Nowadays, renewable bio-based carbon feedstock is highly considered because it offers the intrinsic value of a reduced carbon footprint and an improved life cycle analysis (LCA), in agreement with sustainable development. Hence, the forthcoming materials for daily uses will be generated more and more from biomass [1]. For instance, the production of partially bio-based polyurethanes from vegetable oils and animal fats has largely grown [20,21,22,23]. Most of them are thermosets [24,25,26,27]. However, bio-based TPUs (thermoplastics) have also been investigated and recently developed. Datta et al. [28,29] observed the effect of bio-based components on the chemical, thermal stability, and mechanical properties of TPUs. In their other study, petrochemical and non-chemical polyol-based poly(ether-urethane) were prepared by a solvent-free two-step process, and their properties were compared.

However, according to investigations so far, few studies have considered the application, starting with synthesizing bio-based TPUs using the one-shot method. This study focused on the solvent-free one-shot synthesis of TPUs for fiber spinning containing biomass. In addition, it was aimed to analyze the physical properties of bio-TPU fibers can be used in terms of industrial applications further from the conventional studies on the synthesis of TPUs. When synthesizing TPUs, the molecular weight of the SS was adjusted while maintaining the equivalence ratio of each segment constant to control HS content. Succinic acid-based bio-polyester polyols (M_n_ = 1000, 2000, and 4000 g/mol) with different molecular weights, 1,4 butanediol (BDO), and 4,4′-methylene diphenyl diisocyanate (MDI) were used. As a comparison group, TPUs were synthesized using petroleum-based polyester polyols containing the same acid as bio-based ones. Then, their structural and thermal properties were analyzed and compared. At this time, the most excellent bio-TPUs of the level of petroleum-based TPU were selected. Finally, the physical properties of the extruded fibers under the respective conditions were comparatively analyzed according to the draw ratio. It can be predicted through the major physical property changes that occur due to the difference in the degree of orientation according to changes in fiber draw ratios [30,31]. This study opens new possibilities for developing and applying environmentally friendly TPUs.

## 2. Materials and Methods

### 2.1. Materials

Bio-based polyester polyols (M_n_ = 1000, 2000, and 4000 g/mol), petroleum-based Polyester polyols, and MDI (CAS No. 101-68-8) were kindly provided by Union Chemicals, Inc. (Gyeonggi, Republic of Korea). Provided polyester polyols were synthesized using 1,3-propanediol (PDO, CAS No. 504-63-2) and succinic acid raw materials (CAS No. 110-15-6). BDO (CAS No. 110-63-4) was used as a chain extender, and it was purchased from Daejung Chemicals and Metals Co., Ltd. (Gyeonggi, Republic of Korea). The catalyst (stannous octoate, CAS No. 301-10-0) and heat stabilizer (triphenyl phosphite, CAS No. 3076-63-9) added to TPU resins were supplied by Sambu Fine Chemical Co., Ltd. (Gimhae, Republic of Korea).

### 2.2. Synthesis of Thermoplastic Polyurethanes

TPUs were synthesized using a solvent-free one-shot polymerization procedure [13]. All polyester polyols were heated in a vacuum oven at 80 °C for 3 h, and MDI was melted in the oven at 60 °C for 2 h. A typical polymerization was carried out as follows:

Each polyester polyol and the chin extender BDO were mechanically stirred and heated to 70 °C in a polytetrafluoroethylene (PTFE) reaction vessel. Approximately 0.2 wt% of heat stabilizer and catalyst were added. Then, the appropriate amount of MDI was slowly added. The synthesis was performed under dry nitrogen to prevent the reaction of the isocyanate groups with air moisture [1]. When the temperature was stabilized to the reaction of polyester polyol and isocyanate, the mixture was continuously stirred at 80 °C, 300 rpm for 1 h until a significant increase in viscosity was detected. The corresponding polymer was then poured into a thin layer of PTFE film and post-heated at 80 °C overnight to ensure the complete reaction of the isocyanate groups, which was further monitored by Fourier transform infrared spectroscopy (FT-IR) [1]. The HS content was calculated as a percentage of the mass of chain extender and isocyanate that make up HS out of the total mass of all starting materials. Table 1 presents the procedure in detail.

### 2.3. Preparation of Thermoplastic Polyurethane Fibers

Using petroleum-based TPU-0 and bio-based TPU-1 based on polyester polyols of the same molecular weight, two types of monofilaments with a diameter of 0.2 mm were prepared. These were prepared through the spin draw yarn (SDY) spinning facility of Korea Textile Development Institute, which can spin and draw simultaneously. During spinning, the extruder and die temperatures were set at about 200 to 210 °C during spinning. In addition, the draw ratios were increased by 4, 5, and 6 times, respectively, by changing the winding speed.

### 2.4. Characterization

Gel permeation chromatography (GPC) analysis was performed to measure number-average molecular weight (M_n_), weight-average molecular weight (M_w_) and polydispersity index (PDI). These were measured using GPC equipment (Ecosec GPC, Tosoh company, Tokyo, Japan). TPUs are soluble in some of the common solvents such as tetrahydrofuran (THF), *N*,*N*-dimethyl formamide, ethyl acetate, and methyl ethyl ketone. Therefore, the sample solution for measurement was prepared by completely dissolving 10 mg of dried polymer in 1 mL of inhibitor-free THF solution at room temperature for 1 day and then filtration with a 0.45 µm PTFE filter.

Biobased content was measured in conformity with the standard of ASTM D6866-20. All samples were used for measurement graphitization. The instrument reported raw percent Modern Carbon (pMC) results for each test sample on the isotopic ratio of ^14^C to ^12^C. These results were then adjusted for the ^13^C to ^12^C isotopic ratio determined by the instrument at the time of the test. The adjusted pMC values were assigned a carbon year and adjusted further using Equation (1) to obtain an uncorrected biobased carbon content:

(1) $$\%\mathrm{Biobased}\;C=\frac{\mathrm{pMC}}{\mathrm{Ref}}\times 100$$

where %Biobased C is the uncorrected biobased carbon in the test sample, pMC is the adjusted percent Modern Carbon result from the instrument, and Ref is the reference percent Modern Carbon for the year in which atmospheric carbon became incorporated into biomass via photosynthesis. The reference value used for the carbon year adjustment was 100 pMC per ASTM D6866-20 [32].

Fourier transform infrared (FT-IR) spectra of the TPU samples were analyzed using an FT-IR spectrometer (Nicolet Continuum, Thermo Scientific Inc., Middlesex, MA, USA) with attenuated total reflection (ATR) mode. The spectra were recorded in the range of 4000–500 cm^−1^ at a resolution of 4 cm^−1^.

Differential scanning calorimeter (DSC) analysis was conducted using a DSC instrument (Q2000, TA Instruments, New Castle, DE, USA). The temperature was between −80 to 230 °C, with a heating rate of 10 °C min^−1^. After 3 min, a cooling scan from 230 to −80 °C was performed at a cooling rate of −10 °C min^−1^ under nitrogen as a purge gas. The second scan was used for interpretation. All samples were taken in about 6 mg each in an aluminum pan. 

Thermogravimetric analysis (TGA) and differential thermal analysis (DTA) were performed using a TGA instrument (Q500, TA Instruments, New Castle, DE, USA). TGA began at 30 to 800 °C at a ramping rate of 10 °C min^−1^ in a nitrogen atmosphere. Sample masses of about 30 mg were used.

The physical properties of TPU filaments were analyzed through the following methods. A tensile strength test was conducted according to the ASTM D638 method, using a universal tensile tester (3345R7809, Instron Inc., Norwood, MA, USA). The stretch recovery was measured by KS K ISO13936-2; the elastic modulus of stretch yarn.

The cross-sectional morphology of TPU filaments was observed by an optical microscope (BX-50, Okamoto, Tokyo, Japan).

## 3. Results and Discussion

### 3.1. Structural Characterisation of Thermoplastic Polyurethanes

The weight-average molecular weight (M_w_), number-average molecular weight (M_n_), and polydispersity index (PDI) of TPU samples are listed in Table 2. The average molecular weight and PDI of TPU-0 and TPU-1 synthesized from petroleum-based and bio-based polyols having the same molecular weight (M_n_ = 1000 g/mol) were almost similar, but TPU-0 had a slightly higher average molecular weight and lower PDI. The M_w_, M_n_, and PDI of the TPU-0 were 121,855 g/mol, 96,155 g/mol, and 1.2, respectively. The fact that the molecular weight of the petroleum-based TPU-0 was higher than that of bio-based TPU-1 may have been due to the higher and more even reactivity of the NCO groups of MDI with −OH groups of BDO or polyol, resulting in the observation of a lower value of PDI [33]. Compared to refined petroleum polyols, polyols derived from natural materials contain impurities such as other organic acids, fatty acids, and water remaining in the fermentation process, so it is judged that it can affect the TPU synthesis process.

Meanwhile, TPU-1, TPU-2, and TPU-3 synthesized from bio-based polyols having different molecular weights showed a higher average molecular weight as the molecular weight of the polyol used was lower, and there was little difference in PDI. That is, it can be seen that the higher the HS fraction of bio-based TPUs, the higher the average molecular weight. That is also consistent with the recently reported study results of Nunoz-Guerra et al. [34]. According to previous studies [35,36,37], the lower molecular weight of polyols, the higher HS content, and the characteristic morphology with interconnected domains. On the other hand, as the molecular weight of the polyols increases, the HS content decreases, and the compatibility with HS is low so that the phase separation occurs easily. The polymer has a morphology with an isolated domain. At this time, it is known that the phase transformation to a linked structure occurred when the HS content was about 30 wt% [12,13]. As a result, TPU-0 and TPU-1 with HS content of 30 wt% or more (Table 1) have relatively interconnected domains so that those thermoplastic properties can be sufficiently expressed. In addition, their M_w_ was sufficient for use in polymer processing (e.g., extrusion or spinning) and application. In addition, Table 2 shows the biomass content values through the biocarbon content measurement of the synthesized TPU samples. Bio-based TPU-1, TPU-2, and TPU-3 showed 51–54% bio content. As a result, it was confirmed that more than 51% of bio-based TPU can be successfully synthesized when synthesized with BDO and MDI using a fully biomass-based polyester polyol. 

**Scheme 1:**
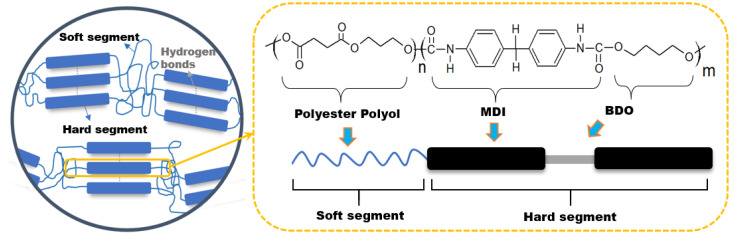
Schematic diagram of the synthetic mechanism of TPU.

Chemical structures of TPUs synthesized using each of the polyols, MDI and BDO, are shown in Scheme 1. The synthesized thermoplastic polyurethanes are made by the exothermic reactions between succinic acid-derived polyester polyol with two reactive hydroxyls (−OH) groups per molecule and MDI with two reactive isocyanate group (−NCO) per molecule. The group formed by the reaction between the two molecules is known as the urethane linkage. After the response, a linear TPU polymer is produced. The chain extender, 1, 4 butanediol in this study, acts as one of the diols to modify the physical properties. 

**Figure 1:**
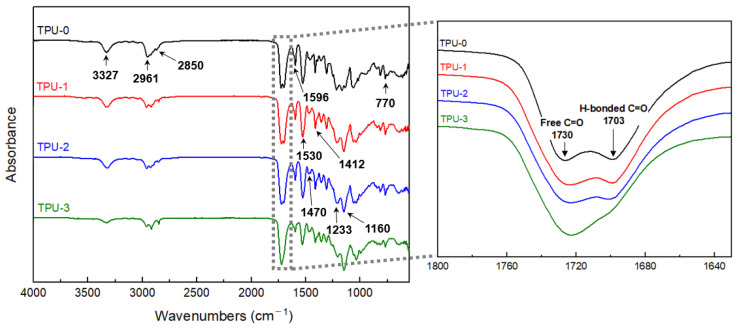
FT-IR spectra of synthesized TPU samples.

The changes in functional groups during polymerization were confirmed using FT-IR analysis, and their spectra are shown in Figure 1. The vibration band at 2270 cm^−1^, which is characteristic of N=C=O, disappears and becomes that of the urethane group, indicating the successful reaction of the −OH group with the N=C=O group [18]. Furthermore, the TPU formation was confirmed by the presence of the vibration at about 3327 and 1530 cm^−1^, attributed to the stretching and bending of the N−H bond of the urethane linkage, respectively. The peak corresponding to the C=O stretching of the urethane group was observed at about 1730 and 1703 cm^−1^ [1]. The appearance of peaks at 1596 and 1470 cm^−1^ reveals the presence of a benzene group. The spectrum also includes absorption bands at 1412 cm^−1^ due to the stretching vibrations of the C−C aromatic benzene ring group [18]. The peak at 1233 cm^−1^ can be attributed to C−O−C urethane vibrations, while the peak at 1160 cm^−1^ is assigned to C−O−C vibrations of the ester groups. The peak at 770 cm^−1^ originates from C−O−O urethane vibrations [38,39,40]. 

In the spectrum of Figure 1, the C=O stretching vibration peak of the urethane group showed absorption bands at different positions depending on the presence or absence of hydrogen bonding. The urethane C=O peak showed an absorption peak at 1703 cm^−1^ due to the mutual hydrogen bonding of HS. On the other hand, the C=O peak, where intermolecular mutual attraction does not act, showed an absorption peak at 1730 cm^−1^. Petroleum-based TPU-0 and bio-based TPU-1, which have the highest HS content, showed high hydrogen bonded C=O peak fractions. In the bio-based TPU samples, it was found that the fraction of the C=O peak at 1703 cm^−1^ forming hydrogen bonds increased as the HS content increased. As the HS content increases, the phase-separation structure of HS changes from an isolated domain to an interconnected domain, so the interphase area between the two phases decreases, making it easier to form hydrogen bonds in the HS domains [12,41]. In addition to the C=O stretching band, the peak fraction increased as the HS content increased according to the gradual reaction of the N=C=O group and the −OH group.

### 3.2. Thermal Characterisation of Thermoplastic Polyurethanes

**Figure 2:**
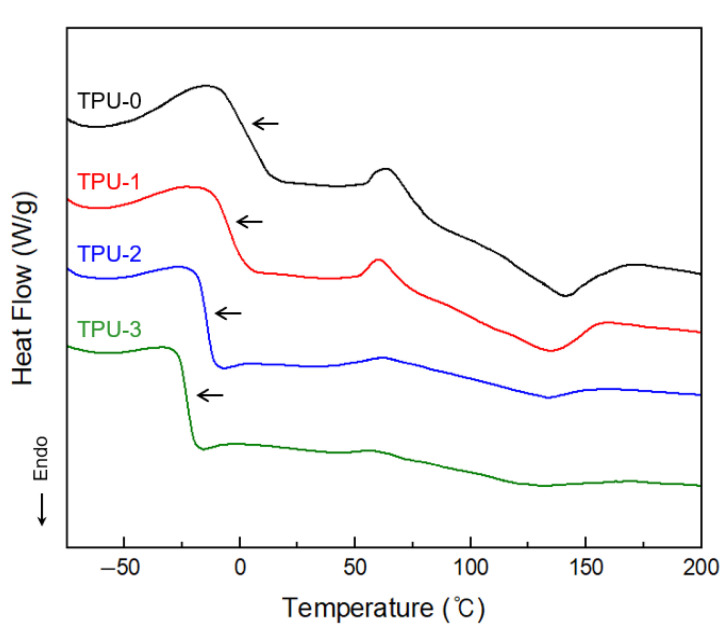
DSC thermograms of synthesized TPU samples.

Figure 2 and Table 3 show the DSC thermograms and numerical values of the samples in which the HS content was changed by varying the molecular weight of petroleum-based and bio-based polyols. The petroleum-based TPU-0 and bio-based TPU-1 samples clearly showed the cold crystallization and glass temperature (T_g,SS_) of SS and the melting temperature (T_m,HS_) of HS. As previously mentioned [12,13], this behavior is considered to be due to the structural phase transition of samples with HS content of 30 wt% or more.

T_g,SS_ of bio-TPUs was increased with HS content. Such deviation of T_g_ was generally considered to result from partial mixing between SS and HS and restriction of rotation of the SS linked to the HS [42,43]. It supports the intuitive view that as the HS content increases, phase mixing increases [44]. As a result, it can be seen that TPU does not achieve an ideal phase-separation structure, so HS and SS exist in a partially mixed structure, and this behavior can be affected by the HS content ratio. This observation was in agreement with the studies by Petrovic et al. [43]. Among bio-TPUs, T_m,HS_ was higher as the HS content increased. As in the previous concept, it was considered that the phase separation between SS and HS increased due to the increase in the HS content, and the amount of SS included in the HS region decreased [45,46]. The broad transition of T_m_, HS of TPUs can also be confirmed by the melting enthalpy (∆H_m, HS_) values in Table 3. 

Petroleum-based TPU-0 and bio-based TPU-1 having the same SS molecular weight show similar DSC curves, but there are slight differences in T_g,SS_ and T_m,HS_ values, respectively. It can be explained by Flory’s Equation (2), which accounts for the melting point change.

(2) $$\frac{1}{T_{m}}-\frac{1}{T{{}^{\circ}}_{m}}=\frac{R}{\Delta H_{u}}\;\ln a$$

where ∆H_u_ is the heat of dissolution per repeat unit, T_m_ and T°_m_ are the melting point in the presence of impurities and the melting point of the pure crystal, respectively, and a is the active concentration of the crystal in the presence of contaminants. Therefore, it can be observed that when a decreases from 1 due to an increase in the number of other components in the copolymer or polymer blend, T_m_ decreases from T°_m_. It shows that bio-based TPU, which contains more impurities in the polymer than petroleum-based TPU, forms less HS due to interference from other components, and T_g_, T_m_ is also lowered.

**Figure 3:**
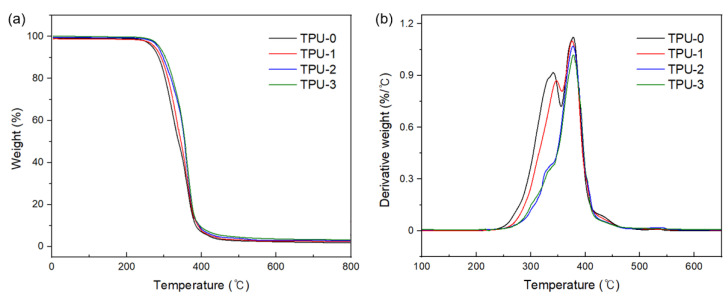
(**a**) TGA and (**b**) DTA thermograms of synthesized TPU samples.

TGA and DTA analyses were carried out to investigate the thermal stability of TPU samples. The results are shown in Figure 3. The characteristic degradation temperatures associated with the thermal degradation are summarized in Table 4. All the TPU samples exhibit similar thermal decomposition behaviors in Figure 3a. The TGA curves show that decomposition occurred in two steps from 300–400 °C [17]. As reported in the literature [47,48], the two-stage thermal decomposition of TPU samples involves the degradation of the HS; urethane linkages in the first stage and SS; polyester polyol in the second stage. In Figure 3b, the range 340–350 °C is associated with the degradation of the HS (T_d1_), and the range 389–390 °C indicates dissociation of the SS (T_d2_) [49]. TPU-0 and TPU-1 showed apparent two-step decomposition behavior, whereas TPU-3 showed less HS decomposition behavior in the first step. Since TPU-3 has a low HS content and forms an isolated domain structure, it is unfavorable to forming urethane groups and hydrogen bonds, so it is expected that the decomposition peak intensity is low. This fact was also consistent with the previous FT-IR structural analysis. 

In addition, the tendency of TPU with lower HS content to have higher T_d2_ and the most residues after thermal degradation was shown in Figure 3a. It is judged that the thermal stability of the high molecular weight TPU is good, but there are few differences in the values. Nevertheless, T_d,5%_ of bio-based TPUs as well as conventional petroleum-based TPU exceeds 300 °C, and fiber production through extrusion processing will be possible.

### 3.3. Mechanical Properties of Thermoplastic Polyurethane Fibers

According to analysis results of the structural and thermal properties, petroleum-based TPU-0 and bio-based TPU having properties suitable for the extrusion process were selected, and the two types of TPU were extruded to produce fibers. They had the same SS molecular weight and had the best phase separation characteristics with an HS content of 30 wt% or more. The composition ratio and phase-separation structure of each segment of TPU greatly affects the physical properties and is mainly affected by the HS content rather than the SS [50,51,52]. 

**Figure 4:**
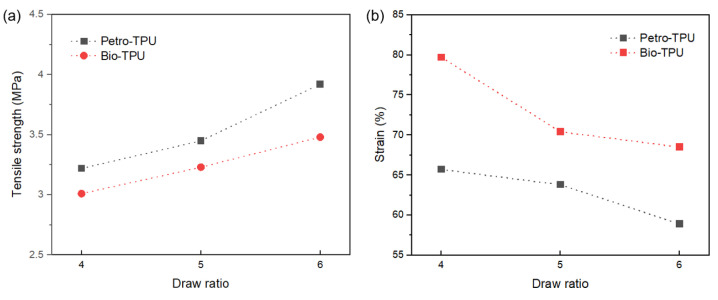
(**a**) Tenacity and (**b**) Strain of Petro-TPU and Bio-TPU according to draw ratios.

The tenacity and strain curves of TPU fibers according to the draw ratios (4, 5, and 6 times) were shown in Figure 4, and the obtained values of tensile strength, elongation at break, and elastic recovery were summarized in Table 5. In addition, all obtained values represent the average value and error obtained by performing the test 10 times. In general, strength and elongation show an inverse correlation [53,54], and this fact was confirmed in both types of TPU fibers, as shown in Figure 4. When the fiber draw ratio was 6 times, the strength of petroleum-based TPU fiber (Petro-TPU) and bio-based TPU fiber (Bio-TPU), respectively, showed maximum values of 3.92 MPa and 3.48 MPa, while the elongation was the lowest at 58.9% and 68.5%. This can be predicted from the previous studies [55,56] because the orientation of HS, as well as SS, develops relatively easily in the stretching direction when the stretching ratio is increased in TPU with a significant HS content. According to H.S. Lee et al. [55], the orientation of rubbery SS occurs primarily according to stretching, and the orientation of the SS applies shear stress to the HS. The long axis of the HS is oriented in the stretching direction, indicating a higher tensile strength value. TPU fiber samples that form interconnected domains with HS content of 30 wt% or more easily form stable HS due to strong hydrogen bonding between HS [12]. In addition, since it results in a significant degree of phase separation, it can be predicted that the orientation behavior of these segments will be more affected. Notably, Petro-TPU showed a tendency to exhibit higher strength and lower elongation than Bio-TPU. As seen in the previous DSC results, the TPU segment changed due to impurities contained in the Bio-TPU.

**Figure 5:**
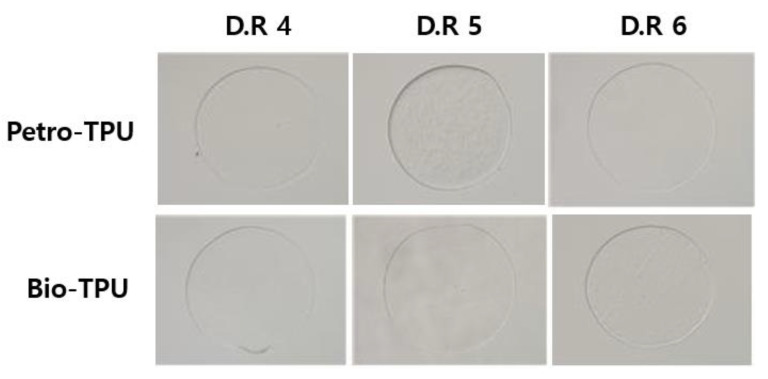
The cross-sectional shape of Petro-TPU and Bio-TPU according to draw ratios (D.R).

In Table 5, Petro-TPU and Bio-TPU fibers with a diameter of about 0.2 mm showed excellent elastic recovery of almost 100%. In general, changes in the internal structure of TPU appear during deformation and relaxation processes. As mentioned above, HS oriented by an external force is relatively stable, whereas SS is flexible. When the external strain is removed, the force to recover to the unoriented state is applied due to the entropy effect of SS. Eventually, the TPU is predicted to be restored to its original form [55]. Figure 5 shows the cross-sectional measurement results of TPU fibers according to the draw ratio. All diameters are (0.2 ± 0.05) mm, and it can be seen that the circular shape is maintained even during stretching, and there is no significant change.

## 4. Conclusions

In this study, bio-based TPU corresponding to the physical properties of existing petroleum-based TPU was synthesized using a succinic acid-based polyester polyol having a different molecular weight through a solvent-free one-shot method. In addition, Bio-TPU was selected with the best characteristics among the synthesized TPUs, and TPU fibers were manufactured through the extrusion process. As a result of GPC and FT-IR analysis, petroleum-based TPU-0 showed the highest molecular weight, and bio-based TPU-1 was most similar with an M_w_ of almost 120,000. In this case, when the M_n_ of the polyol used was as low as 1000, the HS content was characterized as 30% or more. In addition, the increase of the hydrogen-bonded C=O stretching vibration peak indicated the successful synthesis of TPUs. In the results of the DSC and TGA analysis, the characteristics of TPU-0 and TPU-1 were prominent. In both types of TPUs, T_g_ of SS increased, and T_m_ and enthalpy of melting of HS tended to increase. This result was found to be due to the change in the phase separation structure according to the increase in the HS content. The initial decomposition temperature of the synthesized petroleum-based TPU and all bio-based TPUs exhibited sufficient thermal stability for fiber spinning at 300 °C or higher. For this reason, bio-based and petroleum-based TPU fibers were successfully extruded using TPU-0 and TPU-1, which have excellent thermal properties. They exhibited similar physical properties and showed high tensile strength and nearly 100% elastic recovery at a draw ratio of 6 times.

## Figures and Tables

**Table 1:** Composition of TPUs synthesized with various polyols.

Samples	Type of Polyol	M_n_ of Polyol (g/mol)	Polyol:BDO:MDI (Molar Ratio)	HS Content (wt%)
TPU-0	Petroleum	1000	1:1:2	39.0
TPU-1	Biomass	1000	1:1:2	38.8
TPU-2	Biomass	2000	1:1:2	22.3
TPU-3	Biomass	4000	1:1:2	13.3

**Table 2:** Molecular weight and polydispersity of synthesized TPU samples.

Samples	M_w_ (g/mol)	M_n_ (g/mol)	PDI (M_w_/M_n_)	Biobased C (%)
TPU-0	121,855	96,155	1.2	0
TPU-1	120,158	88,215	1.3	54.1
TPU-2	113,738	84,906	1.3	53.0
TPU-3	107,693	81,606	1.3	51.5

**Table 3:** DSC characterization studies of the synthesized TPU samples.

Samples	T_g,SS_ (°C)	T_g,HS_ (°C)	T_m,HS_ (°C)	∆H_m,HS_ (J/g)
TPU-0	−0.67	95.31	135.08	10.80
TPU-1	−4.86	93.57	133.25	9.31
TPU-2	−10.93	89.33	131.22	4.39
TPU-3	−18.54	86.18	130.80	4.04

**Table 4:** TGA characterization studies of the synthesized TPU samples.

Samples	T_5%_ (°C)	T_max%_ (°C)	T_d1_ (°C)	T_d2_ (°C)	Char Residue at 600 °C (wt%)
TPU-0	300.05	408.22	346.81	385.64	1.1
TPU-1	301.32	408.50	346.68	386.40	1.3
TPU-2	302.84	408.74	348.75	386.09	1.3
TPU-3	302.91	409.18	344.07	388.57	1.5

**Table 5:** Tensile strength, elongation at break, and elastic recovery according to draw ratio (4, 5, and 6 times) of Petro-TPU and Bio-TPU.

Samples	4 Times (Draw Ratio)			5 Times (Draw Ratio)			6 Times (Draw Ratio)		
	Tensile Strength (MPa)	Elongation at Break (%)	Elastic Recovery (%)	Tensile Strength (MPa)	Elongation at Break (%)	Elastic Recovery (%)	Tensile Strength (MPa)	Elongation at Break (%)	Elastic Recovery (%)
Petro-TPU	3.22 ± 0.05	65.7 ± 0.2	100	3.45 ± 0.05	63.8 ± 0.2	100	3.92 ± 0.04	58.9 ± 0.1	100
Bio-TPU	3.01 ± 0.08	79.7 ± 0.3	100	3.23 ± 0.06	70.4 ± 0.3	100	3.48 ± 0.05	68.5 ± 0.2	100

## Data Availability

The data presented in this study are available on request from the corresponding author.
